# Time‐Frequency Fingerprint Analysis in SEEG Source‐Space to Identify the Epileptogenic Zone

**DOI:** 10.1002/acn3.70115

**Published:** 2025-07-01

**Authors:** Yash Shashank Vakilna, Deniz Atilgan, Johnson Hampson, Chinmay Chinara, Takfarinas Medani, Richard M. Leahy, Nuria Lacuey, Samden D. Lhatoo, Sandipan Pati, John C. Mosher, Jay R. Gavvala

**Affiliations:** ^1^ Texas Institute for Restorative Neurotechnologies (TIRN), Department of Neurology, McGovern Medical School University of Texas Health Science Center at Houston Houston Texas USA; ^2^ Ming Hsieh Department of Electrical and Computer Engineering University of Southern California Los Angeles California USA; ^3^ Department of Neurology University of Minnesota Medical School Minneapolis USA

**Keywords:** polymicrogyria, seeg, seizure fingerprint, source localization, time‐frequency decomposition

## Abstract

This case study highlights the application of seizure fingerprint analysis in the source‐space of stereo‐EEG (SEEG) data to accurately localize the epileptogenic zone (EZ) in patients with complex cortical malformations. A 25‐year‐old female with extensive bilateral perisylvian polymicrogyria (PMG) presented with intractable focal seizures. The source‐level analysis performed in Brainstorm using the sLORETA imaging algorithm subsequently showed EZ fingerprint analysis. The patient underwent MR‐guided laser interstitial thermal therapy (LITT), targeting the identified EZ, resulting in postoperative seizure freedom with minimal complications. Extending the analysis from the sensor‐space to the source‐space could further enhance surgical planning and improve outcomes in complex epilepsy cases.

## 1 Introduction

The primary aim of a surgical epilepsy evaluation is to delineate the epileptogenic zone (EZ), defined as “the minimum amount of cortex that must be removed for seizure freedom” [1]. Determination of this area is a complex process and requires a comprehensive evaluation. Intracranial EEG recording, such as stereo‐EEG (SEEG), is often required in MRI‐negative cases, widespread epilepsies, and epilepsies in close proximity to eloquent areas [2].

In a majority of cases, the seizure onset pattern plays a prominent role in guiding therapeutic decision‐making. Low‐voltage fast activity (LVFA) is the most common seizure onset pattern described in SEEG recordings [3, 4, 5]. Removal of the areas generating LVFA has been associated with improved postsurgical outcomes [5, 6, 7, 8, 9, 10, 11]. LVFA, however, may occur in multiple areas simultaneously, and visual discrimination may lead to subjective interpretations, particularly in neocortical epilepsies and malformations of cortical developments [12, 13]. Despite several attempts to objectively delineate the extent of the EZ [14, 15, 16, 17], the most common EZ identification methodologies rely on fast activity alone, which has been shown to be inadequate [18, 19]. Additionally, varying methodologies may provide conflicting results depending on the particular ictal pattern [14, 17, 18, 20, 21].

Grinenko et al. 2018 identified a distinct time‐frequency pattern (named “fingerprint”) to define EZ [12]. This pattern has been found to be homogeneous across patients regardless of the etiology, and the regions involved [22]. The description of the EZ fingerprint, however, was limited to sensor‐level analysis, which is dependent on electrode proximity to the EZ. An inherent limitation of the SEEG approach is insufficient sampling, where electrical sources of interest could lie between the electrodes and may be missed [23]. Utilizing SEEG source‐level analysis can overcome this sampling problem by shedding light onto the regions left uncovered by electrodes.

In this study, we present a novel approach that utilizes the EZ fingerprint proposed to determine the EZ in the source‐space constructed using sLORETA. We applied this approach in a case of complex bilateral malformations, including perisylvian polymicrogyria (PMG) and right temporal periventricular nodular heterotopia (PVNH).

## 2 Case Study

A 25‐year‐old right‐handed woman with known drug‐resistant epilepsy since the age of six and right parietal opercular corticectomy at age 15 presented with episodes of focal aware seizures (FASs) with left hand somatosensory aura (tingling) and focal impaired awareness seizures (FIAS) characterized by staring and ictal pouting occurring one to two per week on average. Her evaluation in the epilepsy monitoring unit (EMU) showed intermittent right parietal slowing and 10 habitual seizures, all with right parieto‐central (C4–P4) onset. MRI showed evidence of bilateral perisylvian PMG and pachygyria as well as PVNH in the right posterior temporal lobe in addition to postsurgical changes in the right parietal opercular region. Magnetoencephalography (MEG) showed discharges in the right superior parietal region superior to the prior resection cavity. Based on the noninvasive evaluation, she underwent SEEG lead implantation predominantly targeting the anatomical abnormality over the right hemisphere.

The visual analysis of SEEG demonstrated two seizure onset patterns corresponding to the two semiologically distinct events. FASs with left‐hand somatosensory aura were characterized by a slow polarizing potential and LVFA in the PMG in the right superior parietal region (SPS 7–10 followed by PLON 6–16 and PLAT 6–8). FIASs were characterized by repetitive spiking onset in the posterior insula within the PMG (PIN 1–6).

After a detailed analysis of the SEEG data and discussion in the surgical conference, MR‐guided laser interstitial thermal therapy (LITT) of the right superior parietal polymicrogyria (including the right postcentral cortex identified above) via a right parietal trajectory and right posterior insular polymicrogyria via a right parieto‐occipital trajectory was performed without any complications. She reported being seizure‐free at 1‐year postoperative follow‐up.

## 3 Methods

### 3.1 SEEG Recording

The patient was implanted with 16 SEEG (electrode model PMT2102‐14‐091/2102‐16‐101) with 238 contact leads, out of which 222 contact leads were used for the analysis in total. SEEG was recorded as a part of the routine clinical phase 2 evaluation workflow. SEEG signals were recorded on a Nihon Kohden (Irvine, CA) EEG machine with a 2 kHz sampling rate. The coordinates of the SEEG electrode contacts were estimated by coregistering the postimplant CT with the preimplant MRI using the FSL software package [24]. The electrode coordinates were then exported as an ASCII file.

### 3.2 EZ Fingerprint Identification

**FIGURE 1:**
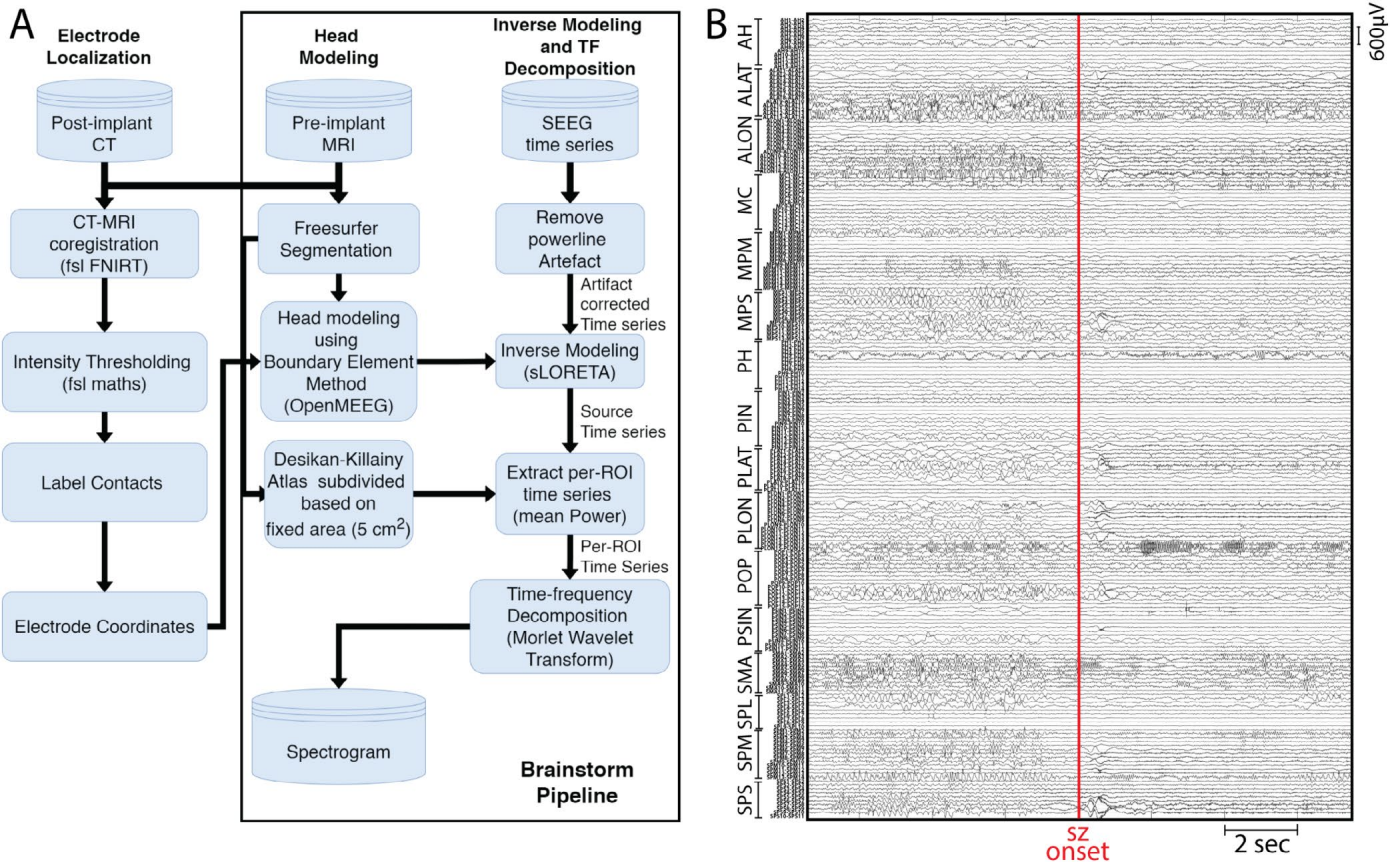
(A) Flowchart illustrating the data‐processing steps involved in computing a spectrogram to identify epileptogenic zone fingerprints on the source‐space. (B) SEEG time series of the analyzed seizure of all the sampled channels in the bipolar montage.

SEEG recordings were visually checked for bad channels, which were excluded from the analysis. As demonstrated in Figure 1, the entire EZ fingerprint identification pipeline was implemented in Brainstorm [25]. The power‐line contamination was removed using a band‐stop notch IIR filter.

During the initial analysis, fingerprint analysis on the sensor level defined in [12] and characterized by a combination of “preictal spikes” and “narrow‐band fast activity” concurrent with “suppression of lower frequencies” was performed, and several electrodes were identified meeting criteria (Figure S1). To further clarify the EZ, source‐level fingerprint analysis was subsequently performed. Cortically surface constrained forward modeling was performed using symmetric BEM [26] with a three‐layer realistic head model (scalp, inner skull, outer skull). The inverse modeling was performed using sLORETA Min‐Norm Imaging [27], further details can be found in Supporting Information S2. Afterward, the Desikan‐Killiany atlas was then used to define multiple regions of interest (ROIs), which were further subdivided to maintain a consistent area of 5 cm^2^ for each ROI. Principal Component Analysis (PCA) was applied to extract the main time series for each ROI (using only the first PCA component). The Morlet wavelet transform (with a wavenumber of 6) was then used to compute the time‐frequency maps of the extracted time series, which were subsequently screened for the presence of EZ fingerprints.

## 4 Results

**FIGURE 2:**
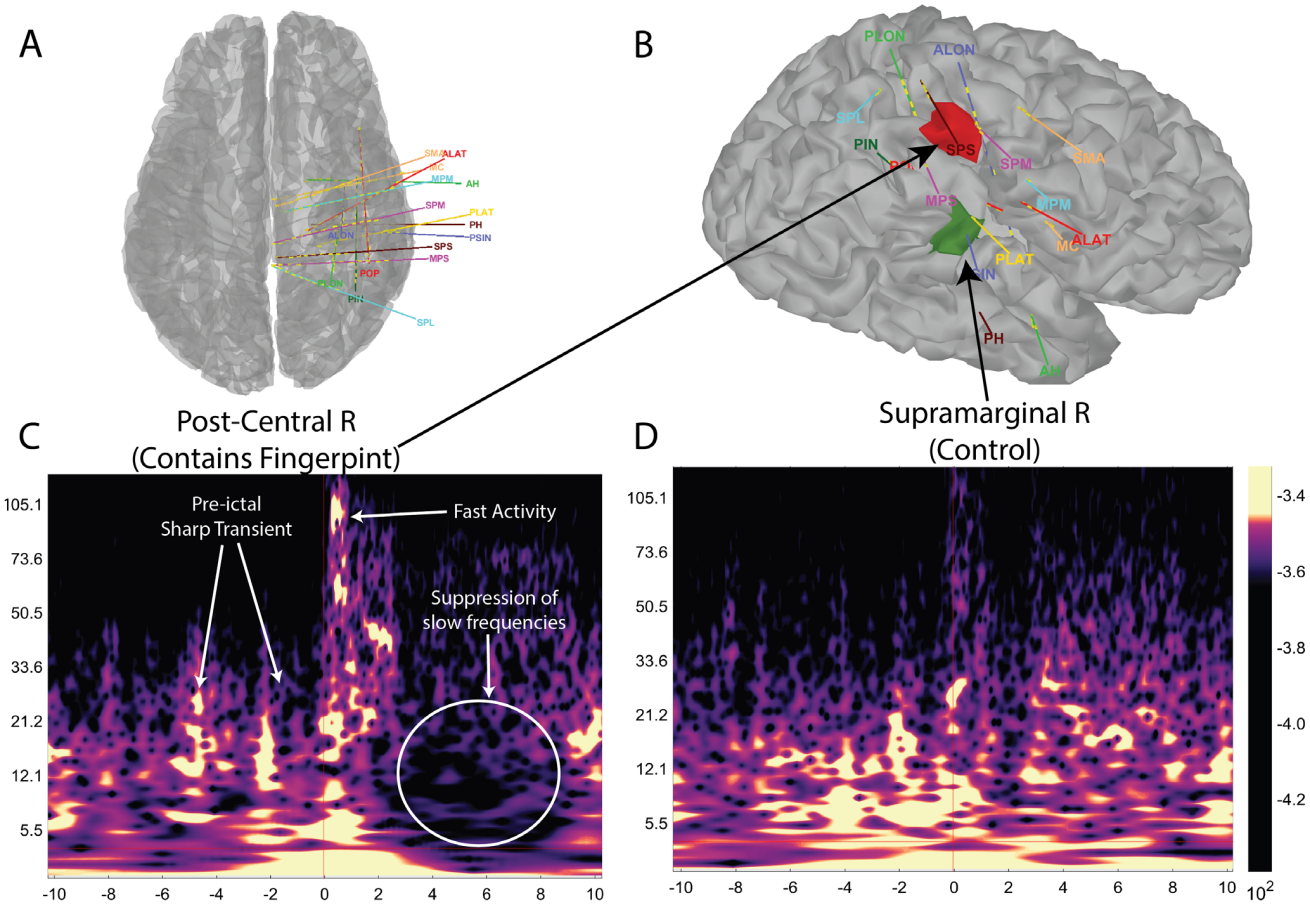
(A) Superior view and (B) Lateral view of cortex with extracted from preimplant MRI showing the SEEG implants. (C) Postcentral R region showing the seizure fingerprint. (D) Supramarginal R region acting as a control.

The SEEG implantation maps are shown in Figure 2A,B; and the time series of the analyzed seizure is shown in Figure 1A. As shown in Figure 2C, we can identify the patch of the cortex with an EZ fingerprint indicative of the EZ. The right postcentral region demonstrated all the properties of the seizure fingerprint. Conversely, other parcellated regions, including the right supramarginal region, did not show the seizure fingerprint.

## 5 Discussion

In this case study, we utilize advanced ictal signal processing techniques to further understand the EZ in a challenging case of lesional epilepsy. After traditional visual analysis of the intracranial ictal patterns, we performed time‐frequency analysis at the sensor level and found the typical fingerprint features characterized by “preictal spikes,” “narrow‐band fast activity” concurrent with “suppression of lower frequencies.” While these findings were well correlated with the conventional visual analysis, they were found in multiple‐depth electrode contacts, limiting the ability to translate to clinical management. Using sLORETA‐based modeling and time‐frequency analysis, we identified the critical brain areas responsible for seizures. This led to a successful surgical outcome using MR‐guided LITT, with the patient experiencing seizure freedom after the procedure. The result highlights the value of this method for guiding surgical decisions, especially in challenging cases where cortical abnormalities are widespread or difficult to assess. Our findings suggest that incorporating source‐level analysis into routine SEEG evaluations can enhance the accuracy of epilepsy surgery planning. Our findings demonstrate the feasibility of utilizing ictal fingerprint analysis in source‐space constructed with SEEG's current density model while using the sLORETA.

SEEG is increasingly being adopted worldwide for presurgical evaluation, and with it, there has been a recognition of the steep learning curve transitioning from a subdural‐based methodology [28]. One of the most challenging problems is knowing when an SEEG electrode represents a true ictal onset pattern versus propagation [29]. While many automated approaches have been developed, many are sensor‐level analyses that either may provide conflicting results or rely on the presence of fast activity, which has been shown to be inadequate in some cases [12]. identified a specific time‐frequency pattern (fingerprint) to define EZ. This pattern has been found to be homogeneous across patients regardless of the etiology and the regions involved [12, 20].

SEEG source localization techniques can theoretically overcome the spatial undersampling problem, which is an inherent problem associated with the recording modality. However, only a handful of studies have been published on using interictal [23, 30, 31] and ictal [31] source localization techniques in SEEG datasets. There is only one study in the SEEG field that analyzed seizure onset with source localization techniques [31]. They analyzed the dominant frequency at seizure onset with both dipole models and current density models. However, they only utilized “fast activity” to delineate EZ rather than the more robust fingerprint analysis.

## 6 Limitations

In this case, the patient had two seizure types and two corresponding seizure onset patterns. We used the sLORETA algorithm to create a current density model of both seizure patterns. However, we only used our novel method for the first seizure type arising from the superior parietal region since there was a clear LVFA onset.

## 7 Conclusion

In this case study, we successfully used EZ fingerprint analysis in the source‐space of SEEG data to localize the EZ in a patient with complex cortical malformations. By extending our analysis to the source‐space, we gained a more precise understanding of the epileptogenic region, which traditional sensor‐level approaches may have missed due to limited spatial coverage. This technique holds the potential for improving surgical outcomes in patients with complex epilepsy and should be explored further in future studies.

## Author Contributions

Conception and design: J.C.M., J.R.G., S.D.L., S.P. Acquisition and data analysis: Y.S.V., D.A., J.H., C.C. Drafting the manuscript: all authors.

## Conflicts of Interest

The authors declare no conflicts of interest.

## Supporting information


Data S1.

## Data Availability

The datasets generated and analyzed during the current study are publicly available in the Zenodo repository at DOI:10.5281/zenodo.14807262 and https://neuroimage.usc.edu/brainstorm/Tutorials/SeizureFingerprinting.
