# Supplementary material for: Time‐Frequency Fingerprint Analysis in SEEG Source‐Space to Identify the Epileptogenic Zone
Source: Ann Clin Transl Neurol. 2025 Jul 1;12(9):1932–6. doi: 10.1002/acn3.70115 (PMC12455889; doi:10.1002/acn3.70115)
Supplement: Supplementary file 1 — Data S1. [file ACN3-12-1932-s001.docx]

**
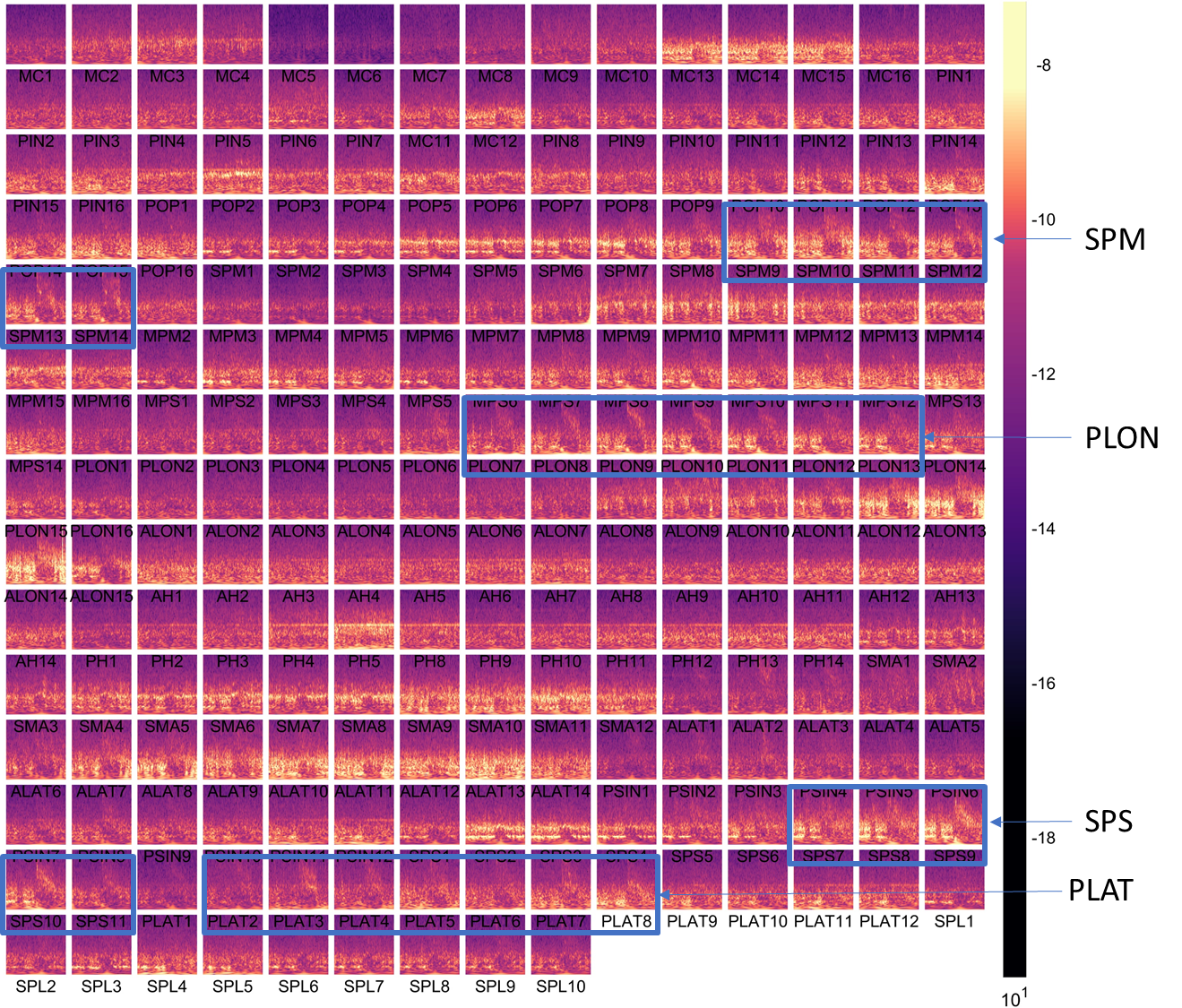
**Supplementary Figure S1

**Supplementary Figure S1:** Seizure Fingerprint – sensor level

**Supplement S2**

Minimum Norm (MN) imaging, also referred to as minimum norm tomography or minimum norm inverse solution, is a specific method used in EEG (electroencephalography) and MEG (magnetoencephalography) for solving the inverse problem of estimating brain activity from scalp measurements. Standardized Low Resolution Brain Electromagnetic Tomography (sLORETA) involves standardizing the current density estimates derived from the Minimum Norm (MN) Imaging solution to address a key limitation of the MN Imaging method.

Here's why this standardization is important:

1. sLORETA Utilizes and Standardizes MN Estimates: sLORETA bases localization inference on standardized values of these current density estimates through a completely different standardization route. This process can be seen as "normalizing" the Minimum Norm images.
2. Achieving Zero Localization Error: A primary benefit of the sLORETA standardization is that it yields images of standardized current density with zero localization error for test dipoles located at voxel positions in the absence of noisy measurements. This accuracy cannot be improved upon. Unlike other previously published instantaneous, distributed, discrete imaging methods for EEG/MEG, sLORETA achieves perfect localization, whereas others produced systematic non-zero localization errors.
3. Improved Performance with Noise: In noisy simulations, sLORETA demonstrates the lowest localization errors compared to the Minimum Norm imaging.

In essence, sLORETA takes the raw current density estimates from the Minimum Norm solution and standardizes them in a specific way that effectively corrects the significant localization errors inherent in the standard Minimum Norm approach, particularly for deep sources, resulting in a more accurate and trustworthy functional imaging method for both EEG and MEG. The standardization process in sLORETA accounts primarily for the variation of actual sources and secondarily for noise variations.

**References:**

Pascual-Marqui RD. Standardized low-resolution brain electromagnetic tomography (sLORETA): technical details. Methods find exp clin pharmacol. 2002 Jan 1;24(Suppl D):5-12.

Pascual-Marqui RD, Esslen M, Kochi K, Lehmann D. Functional imaging with low-resolution brain electromagnetic tomography (LORETA): a review. Methods and findings in experimental and clinical pharmacology. 2002 Jan 1;24(Suppl C):91-5.

Pascual-Marqui RD, Esslen M, Kochi K, Lehmann D. Functional imaging with low resolution brain electromagnetic tomography (LORETA): review, new comparisons, and new validation. Japanese Journal of Clinical Neurophysiology. 2002 Apr 1;30(81):e94.

Supplementary Figure S3

**
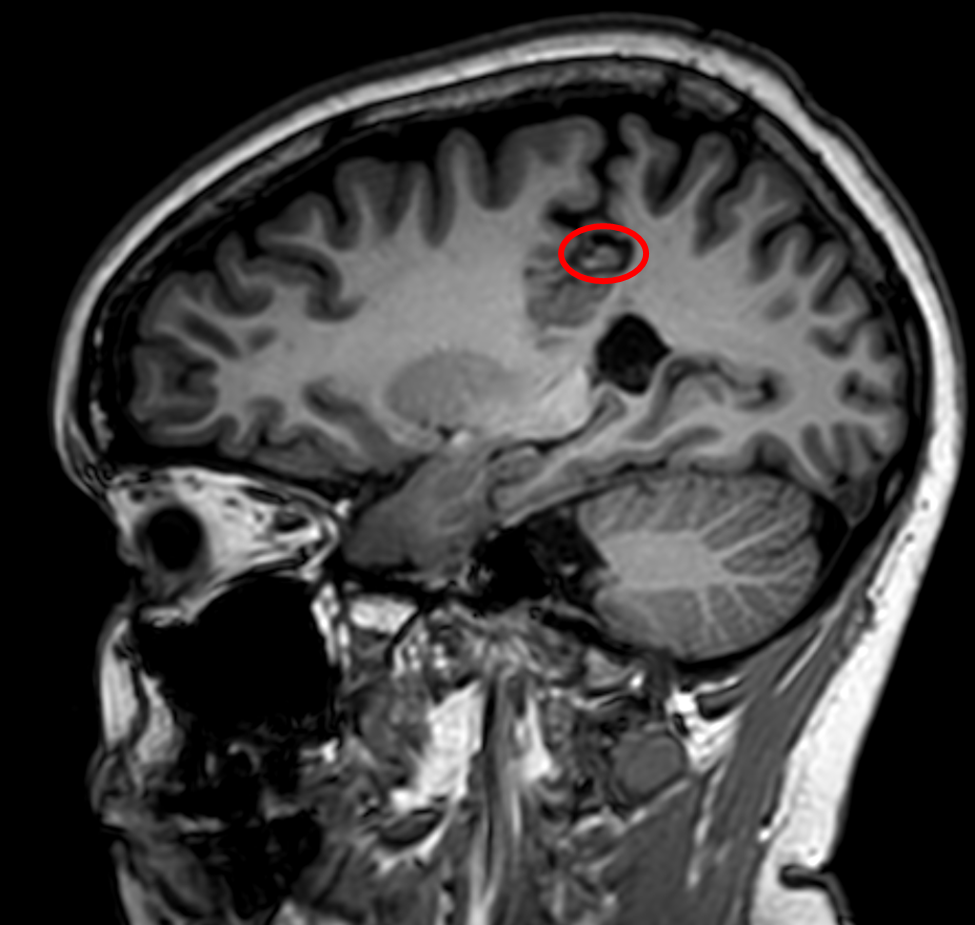

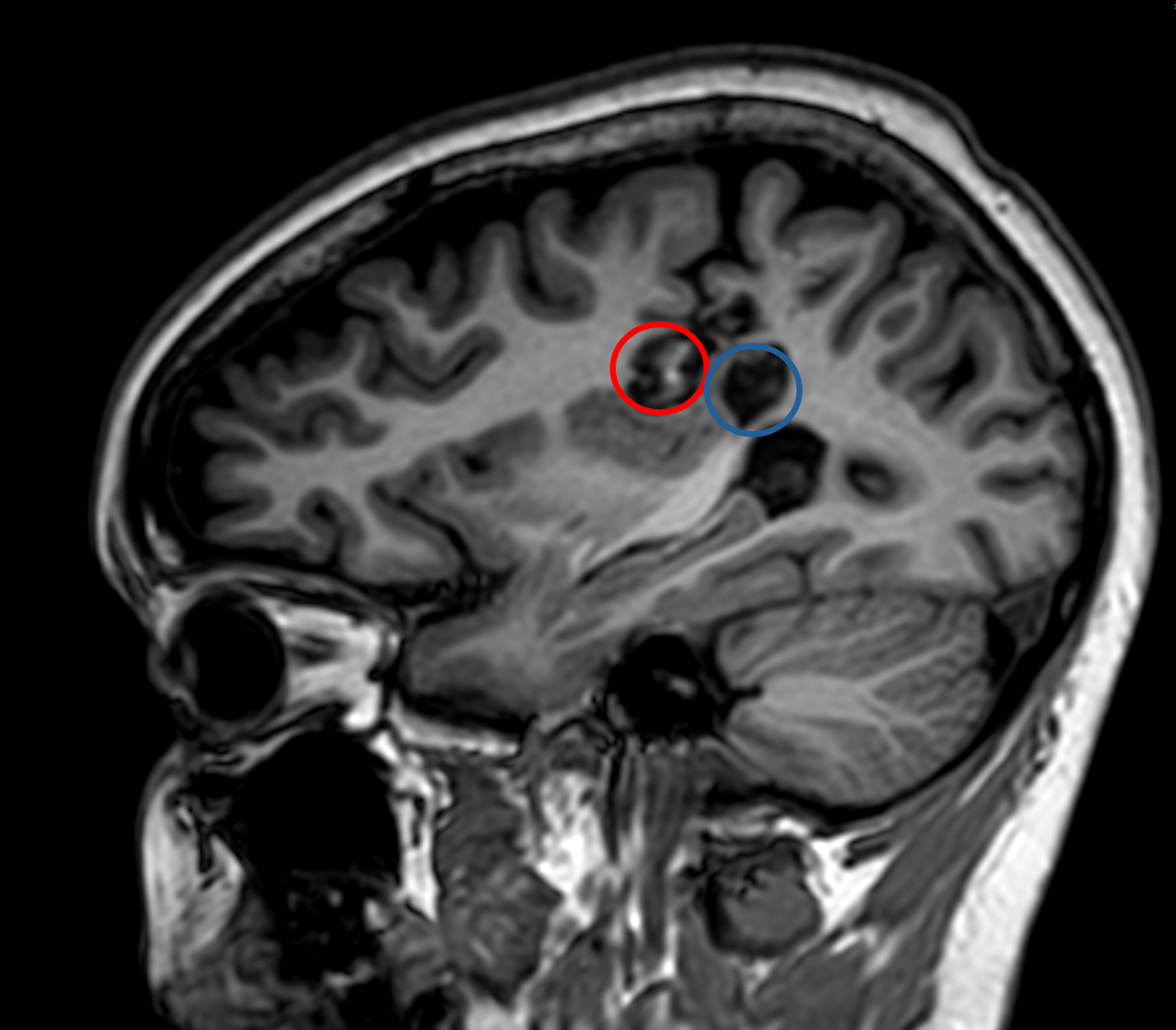
**

**Supplementary Figure S3:** Sagittal view of T1 weighted MRI showing polymicrogyria as well as prior resection cavity and most recent ablation cavity; Red: most recent ablation cavity; Blue: surgical cavity from the prior resection.

**Supplement S4**

All data and scripts used in this study are publicly available as part of the Brainstorm tutorial titled “SEEG Time-Frequency Fingerprint Analysis for Epileptogenic Zone Localization” which can be accessed at this URL: <https://neuroimage.usc.edu/brainstorm/Tutorials/SeizureFingerprinting>.


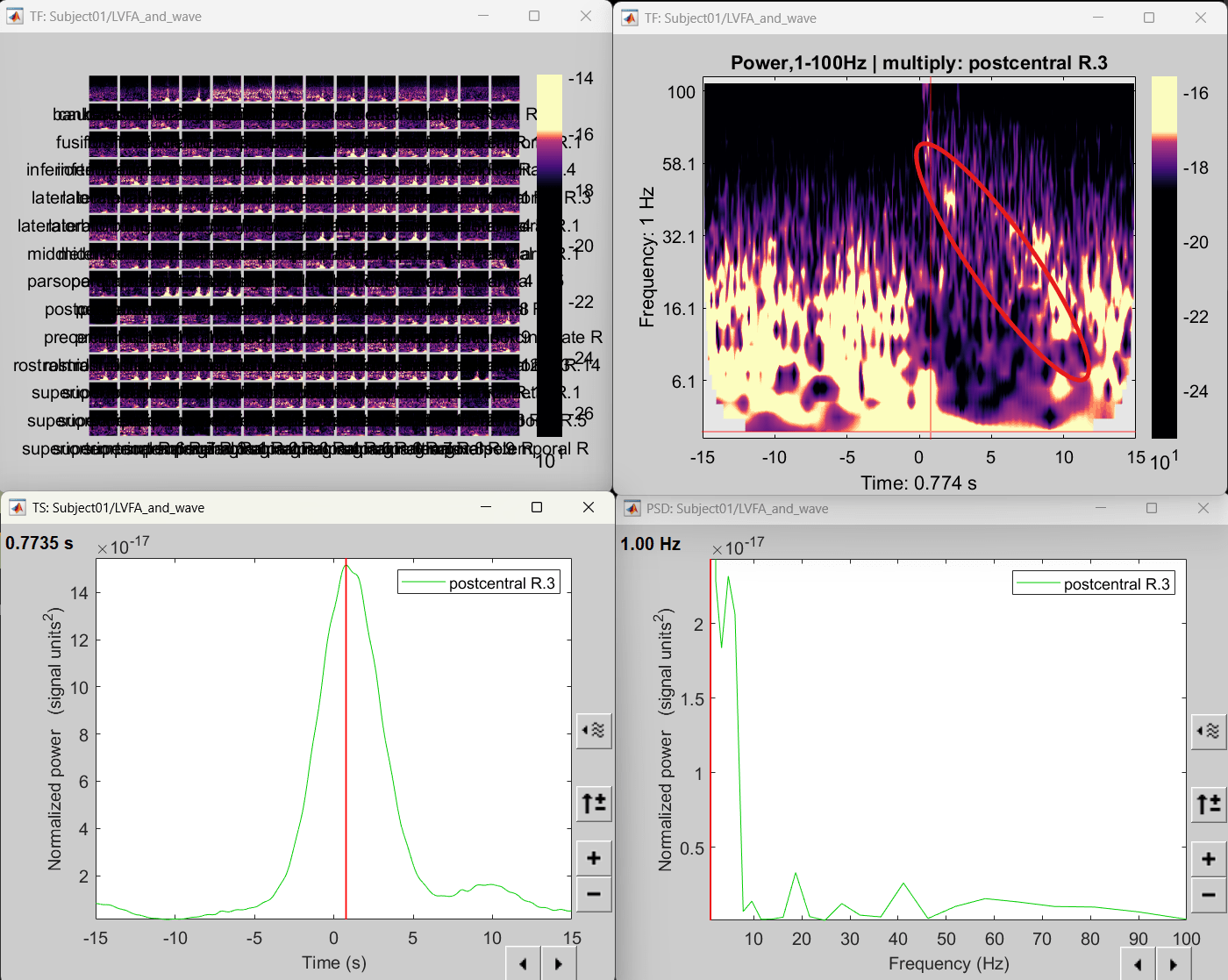


**Supplementary Figure S4:** Epileptogenic zone fingerprint result computed after performing the cortical parcellation using CAT12/SPM12 MATLAB plugin demonstrates qualitatively similar results to those used in the source-space analysis presented in the main manuscript.
